# *In vitro* and *in vivo* evaluation of antifungal combinations against azole-resistant *Aspergillus fumigatus* isolates

**DOI:** 10.3389/fcimb.2022.1038342

**Published:** 2023-01-17

**Authors:** Sana Jemel, Yannick Raveloarisaona, Anne-Laure Bidaud, Elie Djenontin, Aicha Kallel, Jacques Guillot, Kalthoum Kallel, Françoise Botterel, Eric Dannaoui

**Affiliations:** ^1^ Université Paris Est Créteil, Dynamyc, Créteil, France; ^2^ Université Tunis EL Manar, Faculté de médecine de Tunis, Tunis, Tunisie; ^3^ UR17SP03, Centre Hospitalo-Universitaire La Rabta, Jabbari, Tunis, Tunisie; ^4^ Unité de Parasitologie-Mycologie, Service de Microbiologie, Assistance Publique - Hôpitaux de Paris (APHP), Hôpital Européen Georges Pompidou, Paris, France; ^5^ Université de Paris-Cité, Faculté Médecine, Paris, France; ^6^ Dermatology-Parasitology-Mycology, Oniris, Nantes, France; ^7^ Univ. Angers, Univ. Brest, IRF, SFR ICAT, Angers, France

**Keywords:** *Aspergillus fumigatus (A. fumigatus)*, antifungal combination, *Galleria mellonella*, voriconazole, posaconazole, caspofungin, azole-resistance

## Abstract

Azole resistance in *Aspergillus fumigatus* (Af) has become a widespread threat and a major concern for optimal management of patients with invasive aspergillosis (IA). Combination of echinocandins with azoles is an attractive alternative option for the treatment of IA due to azole-resistant Af strains. The aim of this study was to evaluate the *in vitro* and *in vivo* combination of caspofungin (CAS) with either voriconazole (VRZ) or posaconazole (PSZ). *In vitro* interactions were assessed by two methods, and an animal model of IA in *Galleria mellonella* was used for *in vivo* evaluation. Assessment of efficacy was based on larvae mortality. Groups of 10 larvae were infected by 3 clinical strains of Af (azole susceptible, AfS; PSZ resistant, AfR1; VRZ and PSZ resistant strain, AfR2). *In vitro*, combination of CAS and azoles was indifferent against AfS, and AfR2, and a synergy was found for AfR1. When compared to VRZ monotherapy, the combination of VRZ at 4 µg/larva with CAS at 4 µg/larva improved survival of AfR2-infected larvae (p=0.0066). Combination of PSZ at 4µg/larva with CAS at 4 µg/larva improved survival of AfR1-infected larvae compared to CAS (p=0.0002) and PSZ (0.0024) monotherapy. Antagonism was never observed. In conclusion, the combination of caspofungin with azoles is a promising alternative for the treatment of azole resistant strains of Af.

## Introduction

*Aspergillus fumigatus* (Af) remains one of the most potent opportunistic fungal pathogens in humans. It causes a wide range of infections including invasive aspergillosis (IA), a severe condition occurring classically in immunocompromised patients. More recently, other risk factors of IA, such as severe influenza (Schauwvlieghe et al., 2018; Verweij et al., 2020) or severe COVID-19 (Pasquier et al., 2021; Gangneux et al., 2022) have been recognized. IA is associated with high mortality (Latge and Chamilos, 2020; Thompson and Young, 2021), despite effective first line treatment based on azoles (Patterson et al., 2016; Ullmann et al., 2018). Azoles are inhibitors of the sterol 14 α demethylase enzyme, a key step in ergosterol biosynthesis pathway. However, extensive use of azole drugs in the prevention and treatment of fungal infections, and extensive use of fungicides in agriculture, have contributed to the emergence of azole resistance in Af (Lestrade et al., 2019b). Different mechanisms of azole resistance have been reported (Dudakova et al., 2017). The most important is related to changes in the target enzyme by mutation of its gene, but drug efflux has also been reported and resistance can be multifactorial (Dudakova et al., 2017; Rybak et al., 2019). The emergence of azole resistance in Af makes the management of invasive aspergillosis more complex (Verweij et al., 2015). Azole resistance has been associated with treatment failure and excess mortality (Lestrade et al., 2019a; Resendiz-Sharpe et al., 2019).

Therefore, development of alternative treatment options for IA is necessary. The combination of an azole with an echinocandin is one of the therapeutic options (Verweij et al., 2015; Ullmann et al., 2018). The complete evaluation of the efficacy of this kind of combination is an essential step for the validation of the treatment. The aim of this study was to evaluate the *in vitro* and *in vivo* activity of caspofungin (CAS) in combination with voriconazole (VRZ) or posaconazole (PSZ). For *in vivo* evaluation, we used the *Galleria mellonella* model that has proven its contribution to the evaluation of antifungal efficacy for the treatment of IA (Forastiero et al., 2015; Maurer et al., 2015; Jemel et al., 2020; Jemel et al., 2021).

## Materials and methods

### Strains, medium and growth conditions

Three clinical strains of Af, isolated from respiratory samples, were used in the present study. Identification was confirmed by sequencing part of the gene encoding beta-tubulin. The *CYP51A* gene and its promoter had been previously sequenced to determine the mutations involved in azole-resistance (Jemel et al., 2021). We included one azole-susceptible strain (AfS) with a wild type CYP51A sequence, one strain (AfR1) with a G54W mutation and one strain (AfR2) with a L98H point mutation in *CYP51A* in combination with a 34-bp tandem repeat in the promoter (TR34/L98H).

Subcultures were performed on Sabouraud dextrose agar (VWR, Fontenay-sous-bois, France) with chloramphenicol (Sigma-Aldrich, Saint Quentin-Fallavier, France). They were incubated for 7 days at 37°C to obtain sufficient sporulation.

### *In vitro* susceptibility and interaction between caspofungin and azoles

Antifungal susceptibility testing was performed by two methods: the reference microdilution broth technique following the recommendations of the Antifungal Susceptibility Testing Subcommittee of the European Committee on Antimicrobial Susceptibility Testing (EUCAST-AFST), and a concentration gradient strip commercial method (Etest^®^). EUCAST was performed as recommended (Arendrup et al., 2017). For azoles and amphotericin B, minimum inhibitory concentration (MIC) values were determined after 48h of incubation by using a complete inhibition endpoint. For CAS, minimal effective concentration (MEC) endpoints were determined. Gradient concentration strip method (Etest^®^, Biomérieux, Marcy-l’Etoile, France) was performed according to the manufacturer instructions and MICs were read after 48h of incubation.

*In vitro* activity of the combination of CAS with either VRZ or PSZ was first evaluated by the EUCAST reference method modified for a broth microdilution checkerboard procedure (Vitale et al., 2005; Bidaud et al., 2021). Final concentrations ranged from 0.008 to 0.5 µg/mL for CAS, 0.008 to 4 µg/mL for VRZ and PSZ. The final inoculum size in the plates was 1-2.5x10^5^ (CFU) mL^-1^. Microplates were incubated at 37°C and read after 48h of incubation. A growth inhibition endpoint of 50% was used both for the drugs tested alone and in combination. The experiments were performed in triplicate in each of two independent experiments. Data were first analyzed by calculation of the fractional inhibitory concentration index (FICI) interpreted as follow: synergy for FICI ≤ 0.5, no interaction for FICI between 0.5 and 4, and antagonism for FICI > 4 (Odds, 2003). A Bliss independence-based method was also used as previously described (Meletiadis et al., 2005). Two parameters were calculated: the sum (ƩSSI) and the mean (MSSI) of percentages for all statistically significant interactions. Synergy was defined by a ƩSSI >200% and/or a 95% confidence interval of MSSI that did not include 0.

Activity of the combinations was also evaluated by a gradient concentration strip method (Etest^®^) as described previously (Vitale et al., 2005; Bidaud et al., 2021). Briefly, after inoculation of RPMI plates, one strip of VRZ or PSZ were placed on the agar surface for one hour, removed, and a strip CAS was applied exactly on the same position. MICs of the drugs alone and in combination were read after 48h of incubation.

### *Galleria mellonella* inoculation and treatment

#### *Galleria mellonella* infection

Larvae of *G. mellonella* (Kreca^®^ Ento-Feed BV, Ermelo, Netherlands) were used throughout the experiments. In each set of experiments, larvae were randomly distributed in groups of 10 animals.

After culture of the three Af strains, the inoculum was prepared in phosphate-buffered saline containing 0.01% of Tween 20 (PBST), and spore suspensions were adjusted to the required concentration by counting conidia in a hemocytometer. Lethal doses 90% (LD_90_) of each Af strain were previously determined (Jemel et al., 2021). The injection was carried out with 10 µL in the ventral side of the last proleg by using a Hamilton^®^ syringe.

#### Drug preparation

For treatment experiments, VRZ (Vfend^®^ [Pfizer]) and PSZ (Noxafil^®^ [MSD]) were dissolved in 9‰ saline to obtain a stock solution at 10 mg/mL and 18 mg/mL, respectively. Required dosage was obtained by further dilutions in 9‰ saline. For CAS (Cancidas^®^ [MSD]), powder was dissolved in 10 mL of sterile distilled water to obtain a stock solution at 5 mg/mL and further dilutions were performed in 9‰ saline.

#### Caspofungin and posaconazole monotherapy

Groups of 10 larvae were infected by DL_90_ of each Af strains. Two hours after infection, larvae were treated by injection in the ventral side of animal. CAS or PSZ were used at 1, 2, 4 and 8 µg/larva. Larval survival was monitored daily for 7 days. Two control groups were used, the first group consisted of infected larvae inoculated with 9‰ saline at 2h after infection and the second group (to assess toxicity) was only inoculated with the highest doses of CAS or PSZ (8 µg/larva). All experiments were performed two times and results were pooled for analysis.

#### Treatment combination of voriconazole or posaconazole and caspofungin

Solutions of VRZ or PSZ (0.8 µg/µL) and CAS at 0.2, 0.4 and 0.8 µg/µL was obtained by carrying out dilutions in 9‰ saline. At equal volume and before inoculation to larvae, the VRZ solution was mixed with each solution of different concentration of CAS to obtain a combination of VRZ at 0.4 µg/µL and CAS at 0.1, 0.2 or 0.4 µg/µL.

After infection by the three strains of Af, a volume of 10 µL of each antifungal combination was injected in the haemocoel of larvae 2h after infection. Two control groups were used, the first group consisted of infected larvae inoculated with 9‰ saline at 2h after infection. The second group (to assess toxicity) was only inoculated with the highest doses of combination (CAS at 4 µg/larva combined with VRZ or PSZ at 4 µg/larva). Three groups were treated by single VRZ, PSZ or CAS at 4 µg/larva to assess the contribution of combination compared to monotherapy. All experiments were performed three times and results were pooled for analysis.

### Statistical analysis

Mortality curves were generated by Kaplan Meier method and compared by the log-rank test. All analyzes were performed using GraphPad Prism V.3.0 software for Windows (GraphPad Software, San Diego, USA). A value of p<0.05 was considered to be significant.

## Results

### *In vitro* antifungal susceptibility to antifungals

The *in vitro* antifungal susceptibility of Af strains determined by EUCAST and Gradient Concentration Strip (GCS) is presented in **Table 1**. Using EUCAST, AfS with a wild type CYP51A sequence was azole-susceptible. AfR1 with a G54W mutation was resistant to PSZ and itraconazole but susceptible to VRZ. AfR2 with a L98H point mutation in *CYP51A* gene in combination with a 34-bp tandem repeat in the promoter (TR34/L98H), was resistant to the three tested azoles. Results obtained by the Etest^®^ method were within +/- 2 log_2_ dilutions comparable to EUCAST values (**Table 1**, **Figure S1**). For CAS, MEC for AfS, AfR1 and AfR2 was 0.25, 0.5 and 0.5 µg/mL, respectively. CAS MIC values determined by Etest^®^ were systematically lower than EUCAST MEC values.

**Table 1:** *In vitro* susceptibility of the three Af strains.

Antifungal and method	MIC (µg/mL) against		
	AfS	AfR1	AfR2
Amphotericin B			
EUCAST	1	1	0.5
Etest^®a^	0.25	0.5	0.25
Itraconazole			
EUCAST	0.25	>4	>4
Etest^®^	0.5	>32	32
Voriconazole			
EUCAST	0.25	0.5	2
Etest^®^	0.125	0.125	1
Posaconazole			
EUCAST	0.06	>4	0.5
Etest^®^	0.06	>32	0.5
Caspofungin			
EUCAST	0.25	0.5	0.5
Etest^®^	0.032	0.008	0.032

a GCS was determined at 48h for AfS and AfR1, and at 24h for AfR2

### *In vitro* activity of antifungal combinations

When VRZ was combined with CAS, no interaction was observed between the two drugs by FICI (**Table 2**). The lowest FICI for the combination was 1.01, 0.75 and 1.25 for AfS, AfR1 and AfR2, respectively. Bliss analysis showed a synergistic interaction for AfR1 (ƩSSI >200% and 95% CI of MSSI did not include 0), but no interaction for AfS and AfR2 (**Table 3**). Antagonism was not detected for any of the strains.

**Table 2:** *In vitro* interaction between CAS and VRZ by checkerboard.

Isolate	MIC (µg/mL) of drug alone		MIC (µg/mL) of drug in combination		Lowest FICI for the combination	
	CAS	VRZ	CAS	VRZ	CAS/VRZ	Interaction
AfS	1	0.25	0.0156	0.25	1.0156	I
AfR1	1	0.5	0.25	0.25	0.75	I
AfR2	1	2	0.25	1	1.25	I

MIC, Minimal Inhibitory Concentration; FICI, Fractional Inhibitory Concentration Index; CAS, caspofungin; VRZ, voriconazole; I, no interaction.

When PSZ was combined with CAS, no interaction was observed between the two drugs by FICI (0.51) for AfS and AfR2 (**Table 4**). Due to the high level of PSZ resistance (high off-scale MIC), FICI was not computable for AfR1. By Bliss analysis, a synergistic interaction was observed for AfR1, but no interaction for AfS and AfR2 (**Table 3**). No antagonism was observed. Combinations were also evaluated by Etest^®^ (**Figure S2**, **Figure S3**). Combinations were indifferent against all strains (**Table S1**, **Table S2**). There was no antagonism.

**Table 3:** *In vitro* interaction between CAS and PSZ by checkerboard.

Isolate	MIC (µg/mL) of drug alone		MIC (µg/mL) of drug in combination		Lowest FICI for the combination	
	CAS	PSZ	CAS	PSZ	CAS/PSZ	Interaction
AfS	1	0.125	0.0156	0.0625	0.5156	I
AfR1	1	8	ND	ND	ND	ND
AfR2	1	1	0.015625	0.5	0.5156	I

MIC, Minimal Inhibitory Concentration; FICI, Fractional Inhibitory Concentration Index; CAS, caspofungin; PSZ, posaconazole; I, no interaction. ND, not determined (all MICs > maximum).

**Table 4:** *In vitro* interaction between CAS and azoles evaluated by a Bliss independence-based model.

Isolate	CAS/VRZ combination			CAS/PSZ combination		
	ƩSSI	MSSI (95% CI)	Interaction	ƩSSI	MSSI (CI 95%)	Interaction
AfS	50.3%	3.9% (-2.3;10.0)	I	185.5%	23.2% (-2.6;49.0)	I
AfR1	747.5%	37.4% (30.1;44.6)	S	1195.3%	36.2% (30.3;42.2)	S
AfR2	89.5%	6.9% (-4.3;18.1)	I	-12.7%	-4.2% (-23.2;14.7)	I

ƩSSI, Sum of statistically significant interactions; MSSI, Mean of statistically significant interactions; 95% CI, Confidence interval at 95% level; CAS, caspofungin; VRZ, voriconazole; PSZ, posaconazole; S, synergy; I, no interaction.

### Evaluation of caspofungin monotherapy in *Galleria mellonella*

**Figure 1:**
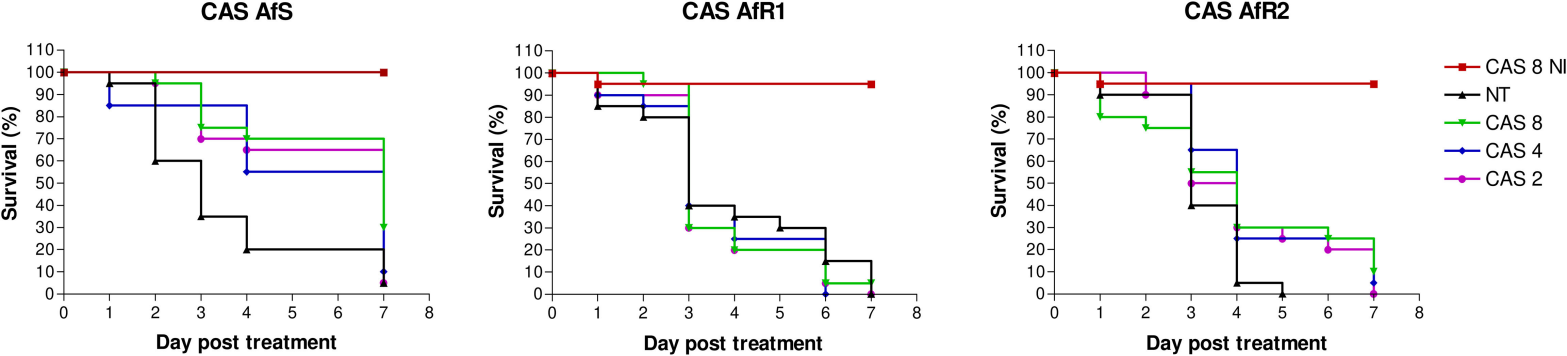
Survival curves of groups of *G. mellonella* larvae inoculated with AfS (left), AfR1 (middle), and AfR2 (right) and treated with caspofungin at 8, 4 or 2 µg/larva after 2h of infection. NI: non infected larvae treated with the highest doses of caspofungin (8 µg/larva). NT: infected larvae and inoculated with 10 µL of saline water. CAS, caspofungin.

For control groups, without treatment, the mortality was at least 95% by day 7, with a median survival time of 3 days for AfS and AfR1 and 3.5 days for AfR2 (**Figure 1**). In AfS-infected groups, CAS at 2, 4, and 8 µg/larva significantly increased the survival during the 7 days of experiment (p=0.0064, 0.017 and 0.0009, respectively). There was no difference in term of efficacy between the different doses of CAS. For AfR1-infected larvae, CAS did not provide any significant improvement in survival with a median survival time of 3 days. For AfR2-infected larvae, only CAS at 4 µg/larva significantly decreased the mortality when compared to the untreated control group (p=0.02).

### Evaluation of posaconazole monotherapy in *Galleria mellonella*

**Figure 2:**
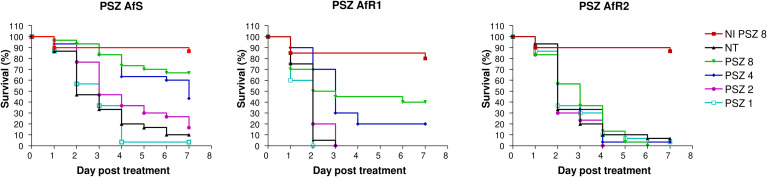
Survival curves of groups of *G. mellonella* larvae inoculated with AfS (left), AfR1 (middle) and AfR2 (right) and treated with posaconazole at 8, 4, 2 or 1 µg/larva after 2h of infection. NI: non infected larvae treated with the highest doses of posaconazole (8 µg/larva). NT: infected larvae and inoculated with 10 µL of saline water. PSZ, posaconazole.

For each strain, efficacy of PSZ at 1, 2, 4 and 8 µg/larva was evaluated (**Figure 2**). Mortality by day 7 in untreated larvae was 90%, 100% and 90% for AfS, AfR1 and AfR2, respectively. Treatment at 4 µg/larva increased survival for AfS (p=0.0004) and AfR1 (p<0.0001) but not for AfR2-infected larvae (p=0.41). In AfS- and AfR1-infected larvae the rate of survival was dose dependent. Although PSZ improved survival compared to untreated controls for both AfS and AfR1-infected larvae, the drug was more effective in AfS than in AfR1-infected larvae. Survival at day 7 was 10%, 50% and 70% for AfS-infected larvae while it was 0%, 20% and 40% for AfR1-infected larvae after PSZ treatment at 2, 4 and 8 µg/larva, respectively. Moreover, median survival for AfS infected larvae and treated with PSZ at 8 and 4 µg/larva was more than >7 days and 7 days compared to 2.5 and 3 days for AfR1-infected larvae.

### Evaluation of combination of voriconazole with caspofungin in *Galleria mellonella*

**Figure 3:**
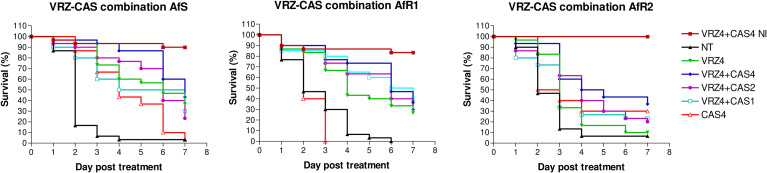
Survival curves of groups of *G. mellonella* larvae inoculated with AfS (left), AfR1 (middle) and AfR2 (right) and treated with monotherapy of voriconazole at 4 µg/larva or combination of voriconazole 4 µg/larva and caspofungin at 4, 2 or 1 µg/larva respectively, after 2h of infection. NI, non-infected larvae treated with combination of voriconazole at 4 µg/larva and caspofungin at 4 µg/larva. NT: infected larvae and inoculated with 10 µL of saline water. VRZ, voriconazole; CAS, caspofungin.

For each isolate, larvae infected with LD_90_ were treated with VRZ at 4 µg/larva combined with CAS at 1, 2 or 4 µg/larva at 2h post infection (**Figure 3**). Mortality, in untreated control groups was at least 95% at day 7 post infection. CAS monotherapy was effective only for AfS-infected larvae (p<0.0001). VRZ monotherapy significantly increased survival of AfS (p<0.0001), AfR1 (p<0.0001) and AfR2-infected groups (p=0.02) compared to untreated group. Nevertheless, the efficacy was better against AfS and AfR1 (survival of 35% and 30%, respectively) than against AfR2 (survival of 10%). The combination of VRZ (4 µg/larva) with CAS (4 µg/larva) significantly increased the survival of AfS (p<0.0001), AfR1 (p<0.0001) but not AfR2-infected larvae (p=0.25) compared to CAS monotherapy at 4 µg/larva. When compared to VRZ monotherapy, the combination (VRZ4 + CAS4) improved survival of AfR2-infected larvae (p=0.0066), but not of larvae infected by AfS (p=0.24) or AfR1 (p=0.28). At a lower concentration, CAS at 1 and 2 µg/larva combined with VRZ at 4 µg/larva did not increase the survival for any of the strain, when compared to VRZ monotherapy.

### Evaluation of combination of posaconazole with caspofungin in *Galleria mellonella*

**Figure 4:**
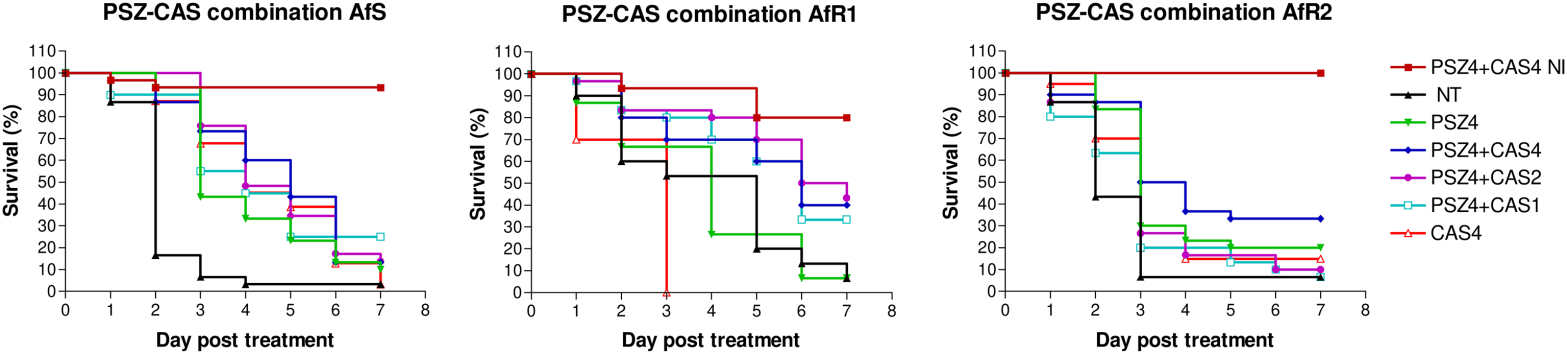
Survival curves of groups of *G. mellonella* larvae inoculated with AfS (left), AfR1 (middle), and AfR2 (right) and treated with monotherapy of posaconazole at 4 µg/larva or combination of posaconazole 4 µg/larva and caspofungin at 4, 2 or 1 µg/larva respectively, after 2h of infection. NI: non infected larvae treated with combination of posaconazole at 4 µg/larva and caspofungin at 4 µg/larva. NT: infected larvae and inoculated with 10 µL of saline water. PSZ, posaconazole; CAS, caspofungin.

For each Af strain, larvae were infected by LD_90_ and treated after 2h by PSZ at 4 µg/larva monotherapy or combined with CAS at 1, 2 or 4 µg/larva (**Figure 4**). At day 7 post-infection, the mortality in the untreated control groups was >90%. Treatment by CAS at 4 µg/larva significantly improved survival for AfS (p<0.0001), but not for AfR1 (p=0.02) or AfR2-infected larvae (p=0.07) compared to untreated group. PSZ at 4 µg/larva significantly improved survival only for AfS (p<0.0001) and AfR2 (p=0.0018) but not for AfR1-infected larvae (p=0.78) compared to the untreated controls. Combination of PSZ at 4 µg/larva and CAS at 4 µg/larva improved survival only for AfR1-infected larvae compared to CAS (p=0.0002) and PSZ (p=0.0024) monotherapy.

## Discussion

In the present study we found very weak *in vitro* interactions between caspofungin and azoles by checkerboard and by agar diffusion. A synergistic interaction was only found for one of the resistant strains (AfR1 resistant to ITZ and PSZ, susceptible to VRZ) when data were analyzed by a Bliss independence-based mathematical model. It has to be noticed that, although *in vitro* testing of antifungal combinations against filamentous fungi are very useful, the techniques are not well standardized, and interpretation of the results is sometimes complicated. Indeed, for azoles-echinocandins combinations, both synergistic and additive effect, depending on the study endpoint and the mathematical definitions for the drug interaction effect, have been reported (Dannaoui et al., 2004; Cuenca-Estrella et al., 2005; Meletiadis et al., 2005; Philip et al., 2005; Jeans et al., 2012; Planche et al., 2012; Seyedmousavi et al., 2013; Mavridou et al., 2015; Raffetin et al., 2018).

For these reasons, in addition to *in vitro* studies, we used an *in vivo* model to assess the combinations. This model was previously used and validated for the evaluation of treatment of aspergillosis (Forastiero et al., 2015; Maurer et al., 2015; Jemel et al., 2020; Jemel et al., 2021). In a first set of experiments, monotherapies were tested at different dosages to assess their efficacy and to determine the optimal dosage for combination studies. VRZ was previously tested in the same model (Jemel et al., 2021), and it was shown that efficacy was correlated to *in vitro* susceptibility, and that a dosage of 4 µg/larva would be suitable for combination experiments. In the present study, we further evaluated CAS and PSZ monotherapies. For CAS monotherapy an increased survival was only observed for AfS-infected larvae but without dose dependent efficacy which is in line with a previous animal study (Lepak et al., 2013).

For PSZ monotherapy, a dose-dependent efficacy was observed for AfS and a lower efficacy against the two PSZ-resistant strains. Nevertheless, a certain degree of efficacy was obtained against the PSZ-resistant strains with a paradoxical better efficacy against the strain with a higher MIC. Although *in vitro*-*in vivo* correlation has been reported for PSZ (Lepak et al., 2013; Forastiero et al., 2015), discrepancies between *in vitro* results and *in vivo* efficacy have also been reported previously. For example, Sun et al. (Sun et al., 2022) evaluated the *in vitro* and *in vivo* efficacy of azoles against Af and observed that PSZ improved significantly the survival of *G. mellonella* larvae infected by a PSZ-resistant strain (MIC of 2 µg/mL). Possible explanations for the efficacy of an antifungal against resistant strains could be the use of high dosages or a lower virulence (fitness-cost) of the resistant strains as shown in the study of dos Reis et al. (Dos Reis et al., 2019) in which some PSZ resistant mutants derived from a wild type strain lost their virulence. Nevertheless, in our work, the LD_90_ was determined for the three strains and no difference in term of virulence was seen between AfS, AfR1 and AfR2 (Jemel et al., 2021).

Overall, in the present study, the combination of an azole with caspofungin showed both indifferent and synergistic interactions depending on the strain susceptibility.

When compared to VRZ monotherapy, the combination of VRZ with CAS had a better efficacy for the VRZ-resistant strain (i.e. AfR2) infected larvae. This is interesting, as combination therapy is recommended in cases of azole-resistance (Verweij et al., 2015). In previous studies, both indifferent (MacCallum et al., 2005; Zhang et al., 2014) and synergistic interactions have been reported (Kirkpatrick et al., 2002), but it has to be noticed that most of the studies have been performed with susceptible isolates.

In our study, combination of PSZ at 4 µg/larva and CAS at 4 µg/larva improved the rate of survival in larvae infected by AfR1 (PSZ resistant strain) when compared to CAS or PSZ alone. These observations are supported by a neutropenic murine model of pulmonary invasive aspergillosis in which efficacy was determined using quantitative PCR (Lepak et al., 2013). Combination therapy with CAS and PSZ did not enhance efficacy for PSZ-susceptible isolates. However, the drug combination produced synergistic activity against PSZ-resistant isolates.

## Conclusion

Overall, our results showed relatively weak interactions between azoles and caspofungin against Af *in vitro*. *In vivo*, a better efficacy of the combination compared to the azole monotherapy was obtained only against the azole-resistant isolates. Antagonism was never observed.

## Data availability statement

The original contributions presented in the study are included in the article/**Supplementary Material**. Further inquiries can be directed to the corresponding author.

## Author contributions

Conceptualization, ErD. methodology, SJ, YR and A-LB., formal analysis, SJ YR, A-LB, and ErD. data curation, SJ A-LB, and ErD. writing—original draft preparation, SJ and ErD. writing—review and editing, SJ, YR, A-LB, JG, KK, FB, ErD and EID. supervision, ErD. funding acquisition, FB, ErD, and JG. All authors contributed to the article and approved the submitted version.

## Supplementary material

Supplementary Table 1
*In vitro* interaction between CAS and VRZ by gradient concentration strips

Supplementary Table 2
*In vitro* interaction between CAS and PSZ by gradient concentration strips

Supplementary Figure 1
*In vitro* determination of Minimal Inhibitory Concentrations of five antifungals by gradient concentration strip for the three stains of *Aspergillus fumigatus*: AfS, AfR1 and AfR2. AMB: amphotericin B, ITZ: itraconazole, VRZ: voriconazole, PSZ: posaconazole and CAS: caspofungin.

Supplementary Figure 2
*In vitro* determination of Minimal Inhibitory Concentrations by gradient concentration strips of voriconazole alone, caspofungin alone, and the combination of voriconazole with caspofungin for the three strains of *Aspergillus fumigatus* (AfS, AfR1 and AfR2). VRZ: voriconazole; CAS: caspofungin.

Supplementary Figure 3
*In vitro* determination of Minimal Inhibitory Concentrations by gradient concentration strips for posaconazole alone, caspofungin alone and combination of posaconazole with caspofungin for the three strains of *Aspergillus fumigatus* (AfS, AfR1 and AfR2). PSZ: posaconazole; CAS: caspofungin.
