# Supplementary figures and images for: In vitro and in vivo evaluation of antifungal combinations against azole-resistant Aspergillus fumigatus isolates
Source: Front Cell Infect Microbiol. 2023 Jan 17;12:1038342. doi: 10.3389/fcimb.2022.1038342 (PMC9887171; doi:10.3389/fcimb.2022.1038342)

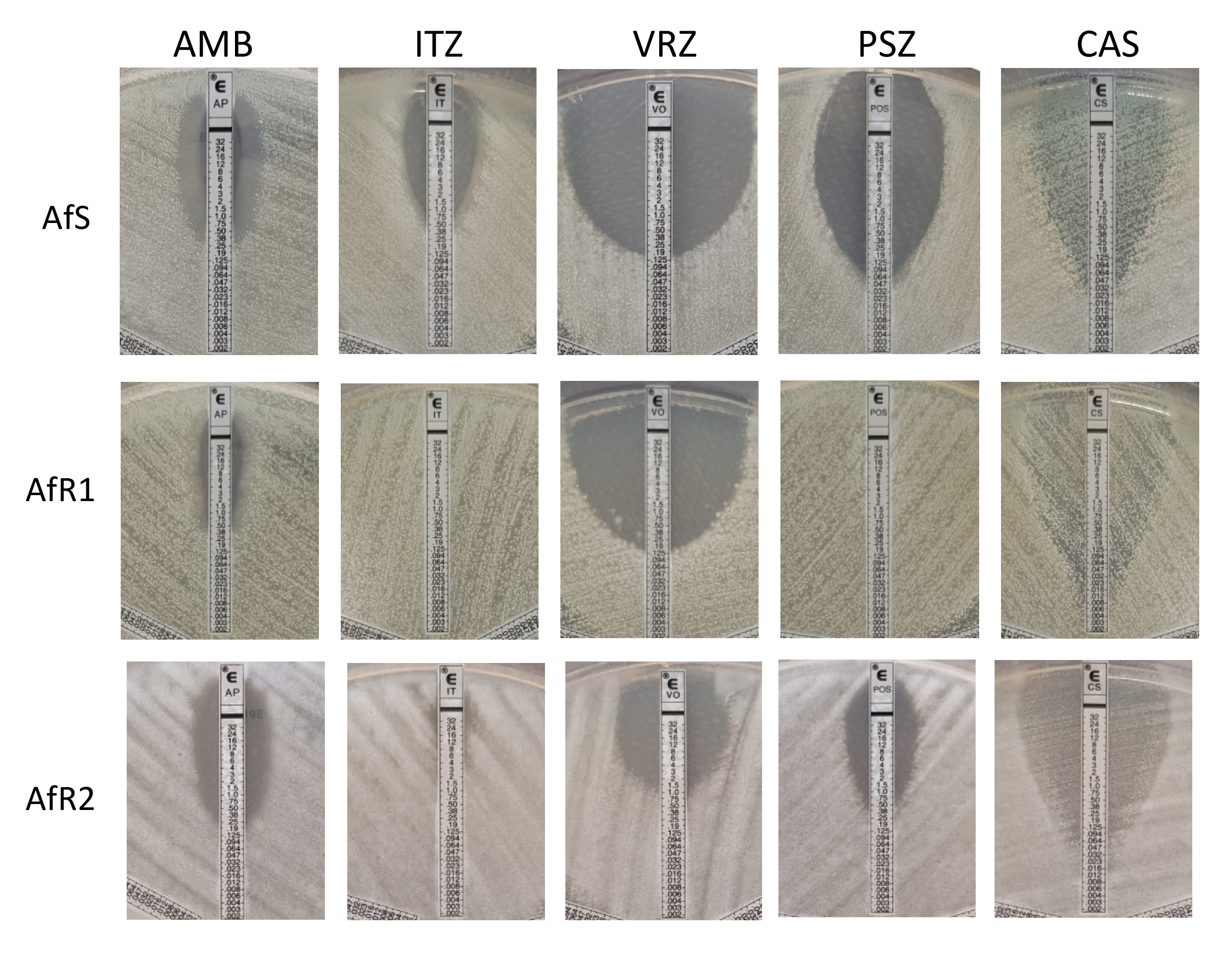

Supplement: Supplementary Table 1 — In vitro interaction between CAS and VRZ by gradient concentration strips [file Image_1.tif]

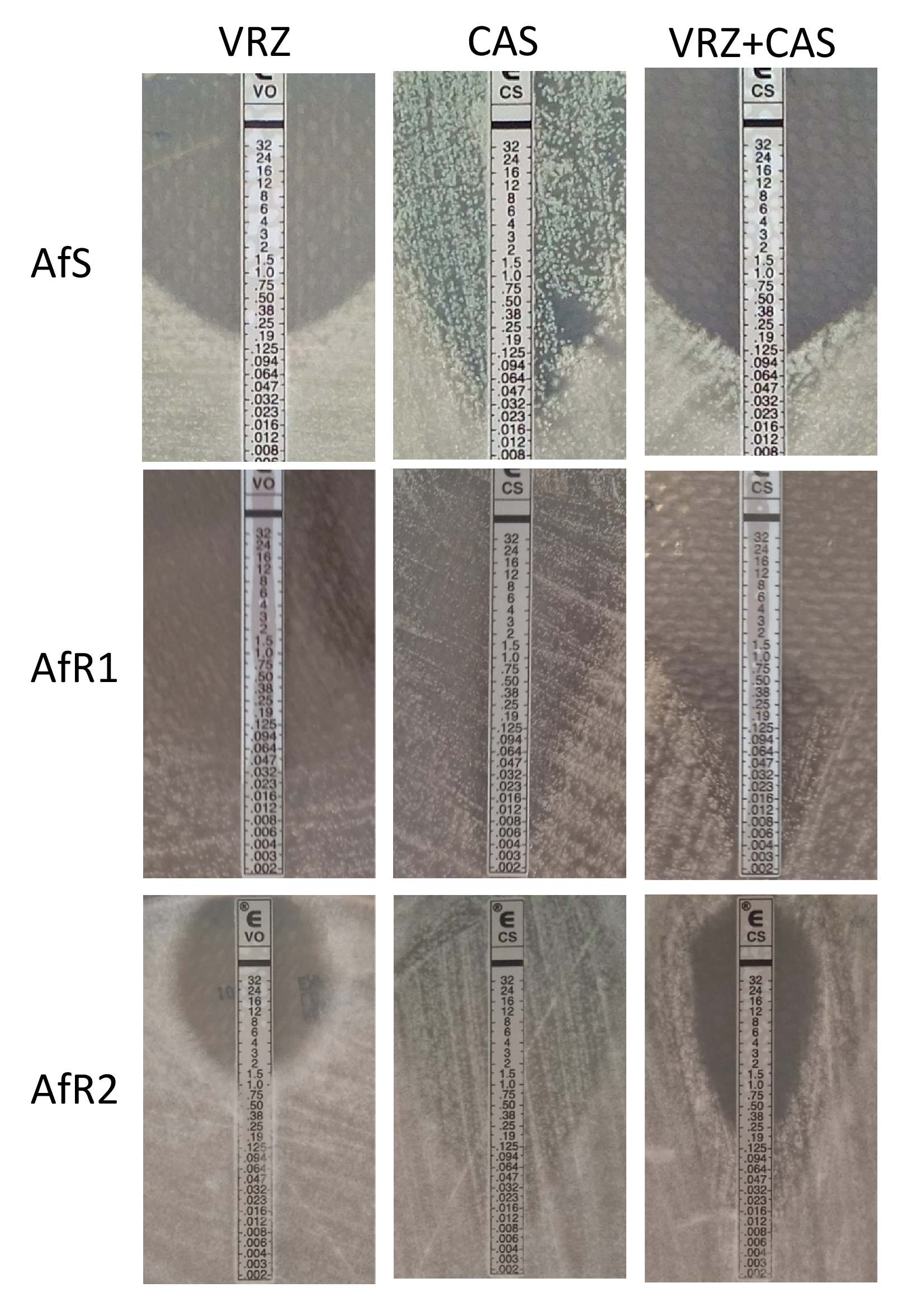

Supplement: Supplementary Table 2 — In vitro interaction between CAS and PSZ by gradient concentration strips [file Image_2.tif]

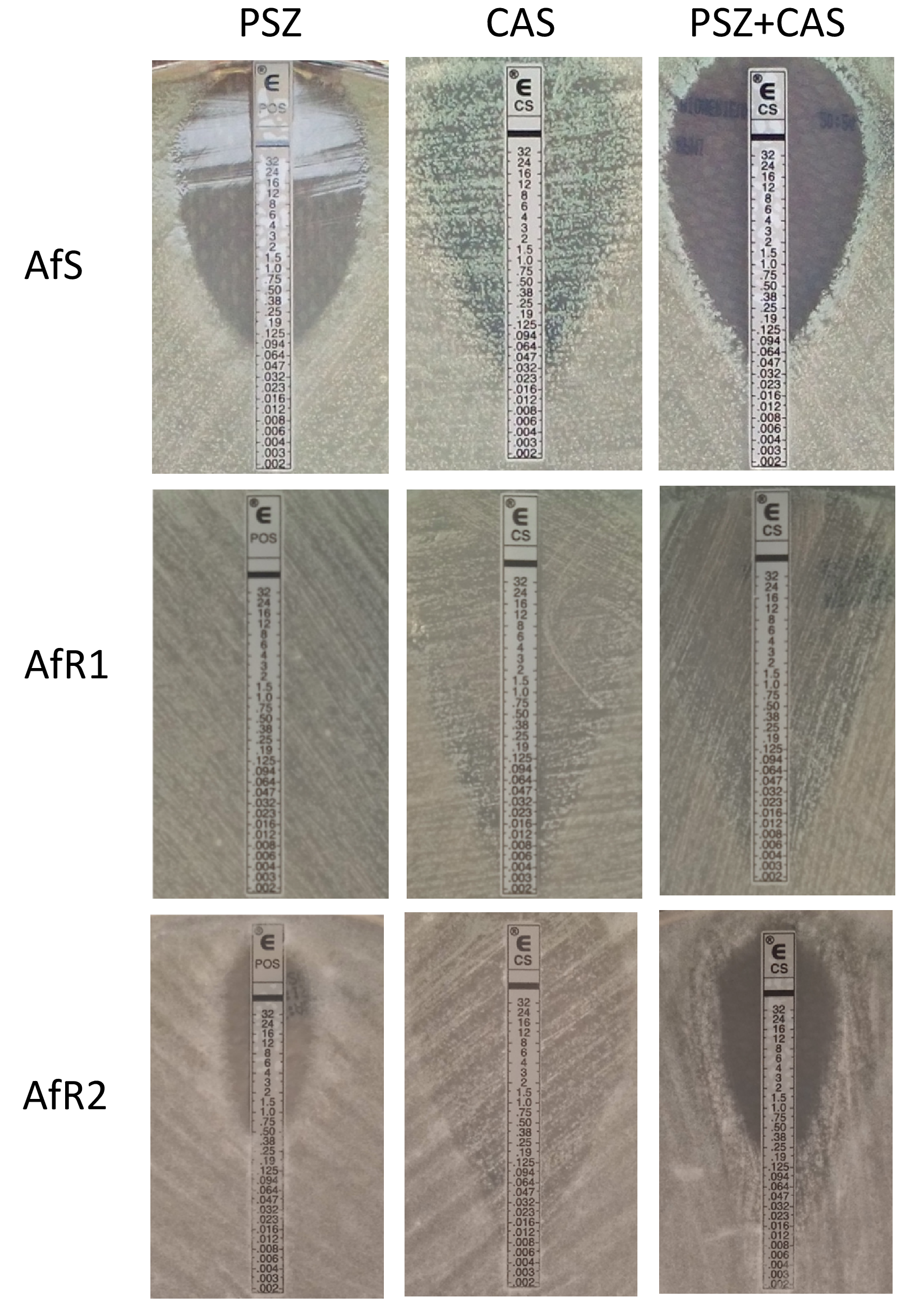

Supplement: Supplementary Figure 1 — In vitro determination of Minimal Inhibitory Concentrations of five antifungals by gradient concentration strip for the three stains of Aspergillus fumigatus: AfS, AfR1 and AfR2. AMB: amphotericin B, ITZ: itraconazole, VRZ: voriconazole, PSZ: posaconazole and CAS: caspofungin. [file Image_3.tif]
