# Supplementary material for: In vitro and in vivo evaluation of antifungal combinations against azole-resistant Aspergillus fumigatus isolates
Source: Front Cell Infect Microbiol. 2023 Jan 17;12:1038342. doi: 10.3389/fcimb.2022.1038342 (PMC9887171; doi:10.3389/fcimb.2022.1038342)
Supplement: Supplementary Figure 2 — In vitro determination of Minimal Inhibitory Concentrations by gradient concentration strips of voriconazole alone, caspofungin alone, and the combination of voriconazole with caspofungin for the three strains of Aspergillus fumigatus (AfS, AfR1 and AfR2). VRZ: voriconazole; CAS: caspofungin. [file Table_1.docx]

**Table S1:** In vitro interaction between CAS and VRZ by gradient concentration strips

| **Isolate** | **MIC (µg/mL) of drug alone** | |  | **MIC (µg/mL) of drug in combination** | | **FICI for the combination** | |
| --- | --- | --- | --- | --- | --- | --- | --- |
|  | **CAS** | **VRZ** |  | **CAS+VRZ** |  | **CAS +VRZ** | **Interaction** |
| AfS | 0.023 | 0,19 |  | 0.023 |  | 1.12 | I |
| AfR1 | 0.004 | 0.032 |  | 0.004 |  | 1.12 | I |
| AfR2 | 0.016 | 1.5 |  | 0.016 |  | 1.01 | I |

MIC: Minimal Inhibitory Concentration; FICI: Fractional Inhibitory Concentration Index; CAS: caspofungin; VRZ: posaconazole; I: no interaction.
