# Supplementary material for: In vitro and in vivo evaluation of antifungal combinations against azole-resistant Aspergillus fumigatus isolates
Source: Front Cell Infect Microbiol. 2023 Jan 17;12:1038342. doi: 10.3389/fcimb.2022.1038342 (PMC9887171; doi:10.3389/fcimb.2022.1038342)
Supplement: Supplementary Figure 3 — In vitro determination of Minimal Inhibitory Concentrations by gradient concentration strips for posaconazole alone, caspofungin alone and combination of posaconazole with caspofungin for the three strains of Aspergillus fumigatus (AfS, AfR1 and AfR2). PSZ: posaconazole; CAS: caspofungin. [file Table_2.docx]

**Table S2:** In vitro interaction between CAS and PSZ by gradient concentration strips

| **Isolate** | **MIC (µg/mL) of drug alone** | |  | **MIC (µg/mL) of drug in combination** | |  | **FICI for the combination** | |
| --- | --- | --- | --- | --- | --- | --- | --- | --- |
|  | **CAS** | **PSZ** |  | **CAS+PSZ** |  | | **CAS +PSZ** | **Interaction** |
| AfS | 0.032 | 0.094 |  | 0.064 |  | | 2.67 | I |
| AfR1 | 0.006 | >32 |  | 0.006 |  | | 1.00 | I |
| AfR2 | 0.016 | 0.5 |  | 0.016 |  | | 1.03 | I |

MIC: Minimal Inhibitory Concentration; FICI: Fractional Inhibitory Concentration Index; CAS: caspofungin; PSZ: posaconazole; I: no interaction.
